# Synthesis and Structure of 2-Hydroxypropyl Methacrylate-Capped Isophorone Diisocyanate and Poly(Propylene Glycol) Urethane Mixtures and the Properties of their UV-Cured Co-Networks with Isobornyl Methacrylate

**DOI:** 10.3390/ma15238586

**Published:** 2022-12-01

**Authors:** Junhao Zhou, Liming Tang

**Affiliations:** Key Laboratory of Advanced Materials of Ministry of Education of China, Department of Chemical Engineering, Tsinghua University, Beijing 100084, China

**Keywords:** polyurethane methacrylate prepolymer, UV curing, solvent-free fabrication, structure, property

## Abstract

Polyurethane acrylate prepolymers with different contents of HIPIH and HIH were synthesized via reacting excessive isophorone diisocyanate (IPDI) with poly(propylene glycol) (PPG) and then end-capping with 2-hydroxypropyl methacrylate (HPMA) in isobornyl methacrylate (IBOMA). After the addition of the photoinitiator PI 1173, the resulting prepolymer resins were irradiated by UV light to form cured materials. The structures of the prepolymers were confirmed by ^1^H NMR, FT-IR, and GPC. SEM analyses proved that no obvious phase separation was observed within the cured sample. As the content of HIH increased, the viscosity of the prepolymers increased slightly. In addition, the gel content, solvent resistance, Shore hardness, Young’s modulus, and the tensile strength of the cured films increased, whereas the elongation at break decreased gradually. The volume shrinkage of the cured samples ranged between 4.5% and 4.8%. DMA analyses showed that the Tgs of the cured samples increased as more HIH structures existed. TGA analyses revealed that the cured samples had high thermal stability. This solvent-free fabrication process was simple, convenient, and controllable. By simply regulating the contents of HIPIH and HIH in the prepolymers, the performances of the cured materials could be adjusted to a wide range.

## 1. Introduction

UV curing technology has the advantages of fast curing, low energy consumption, wide adaptability, low volatile organic chemical emissions (VOCs), room temperature operation, and so on [1,2,3,4]. Its peculiar 5E features (i.e., efficient, energy-saving, economical, enabling, and environmentally friendly) make it a green and popular technology. So far, it has been widely used in coating [5,6], ink [7,8], varnish [9], adhesive [10], 3D printing [11], functional materials [12,13], etc. Under UV irradiation, the curable system instantaneously forms a cross-linking network and turns from liquid to solid within a fraction of a second. In general, the curing system consists of the following three primary components: a photoinitiator (PI), a photosensitive resin (an oligomer or a prepolymer), and a diluent (an active monomer). Additionally, some auxiliaries and pigments are incorporated frequently. According to the different curing mechanisms, UV curing can be roughly divided into the following two types: free-radical UV curing and cationic UV curing. In free-radical curing systems, acrylate raw materials that contain C=C double bonds are mostly selected, and the double bonds are polymerized under the action of a photoinitiator. Cationic curing systems are mostly based on epoxides or vinyl ethers [14], wherein epoxy functional groups are polymerized through ring opening. Compared with cationic curing, free-radical curing is more commonly used [15]. A significant advantage of free-radical curing systems is their fast curing speed, but the problem faced by the system is serious oxygen inhibition [16,17], which leads to incomplete curing and a sticky surface. In addition, larger volume shrinkages produce stress on the substrates and decrease their adhesion ability [18]. The cationic curing system has the considerable merits of being free from oxygen inhibition in the air, having low volume shrinkage, high adhesion (especially for metals), and post-curing (under darkness) [14,19,20]. Common problems with cationic curing include slow curing speed (relative to free-radical curing) and superacids produced by the PIs, such as iodonium and sulfonium salts, which may cause corrosion to the substrates. Overall, radical UV curing is more mature and advantageous.

A prepolymer is the core component of a curing system, which directly determines the mechanical performance and indicators of the cured samples, such as hardness, modulus, glass transition temperature (Tg), etc. Monomers also play an important role in the properties of the cured materials. They not only adjust the viscosity of curable resins as diluents for the convenience of processing, but also participate in the formation of a cross-linking network after the curing, which has a certain impact on the final performance of the cured samples. The monomers used have low viscosity, a favorable dilution effect, good compatibility with the resin, low volatility and toxicity, etc. The prepolymers used for radical UV curing mainly include polyurethane (meth)acrylates (PUA or PUMA), epoxy acrylates, unsaturated polyesters, etc. Generally, the PUA (or PUMA) prepolymer can be synthesized through the reaction of polyol with excess diisocyanate, after which (meth)acrylate containing hydroxyl is introduced to react with the residual NCO groups, hence, producing a prepolymer with reactive (meth)acrylate groups. PUA and PUMA are investigated extensively because of their alternative structures, excellent chemical resistance, good toughness, flexibility, and adhesion to different substrates [21,22]. For example, Li et al. [23] synthesized a novel polyurethane acrylate prepolymer based on castor oil, which is a renewable raw material. The resulting UV-cured coating had a volume shrinkage of 4.99~8.91%, a tensile strength of 9.87~17.84 MPa, and a Tg of about 60 °C. Cheng et al. [24] developed castor oil-based, high-transparency, silicone-modified UV-cured polyurethane acrylate coatings with outstanding tensile strength and good chemical resistance using castor oil-based polyurethane acrylates and thiol silicone resins. The UV-cured coatings had a transparency of 90~98%, a tensile strength of 10.2 MPa, and an elongation at break of 88.4~162.5%. Noreen et al. [25] prepared microalgal protein-based UV-curing polyurethane acrylate with hexanediol diacrylate precursors and amino acid oligomers. The obtained cured material possessed a Tg of 122 °C, a tensile strength of 19.1 MPa, a modulus of 465 MPa, and an elongation at break of 6%. Despite the many advances in the past decade, UV-cured resins with comparatively low viscosities, small volume shrinkages, high mechanical strengths, and high Tgs are still being investigated.

The functionality of both the prepolymer and monomer has a significant influence on the materials’ performances [26]. In this paper, we aimed to develop novel free-radical UV-curable PUMA resins that possess the abovementioned excellent performances by reacting isophorone diisocyanate (IPDI) with poly(propylene glycol) (PPG) and 2-hydroxypropyl methacrylate (HPMA) using isobornyl methacrylate (IBOMA) as the reactive diluent and PI 1173 as the photoinitiator. By simply adjusting the feeding formula, prepolymers with different contents of HIPIH (an abbreviation for the ideal structure of HPMA-IPDI-PPG-IPDI-HPMA) and HIH (an abbreviation for the ideal structure of HPMA-IPDI-HPMA) were fabricated. The prepolymer resins all had low viscosities, and their UV-cured materials exhibited adjustable mechanical properties and prominent thermal stabilities, along with the desired solvent resistances and relatively low volume shrinkages. This one-pot reaction to generate UV-curable prepolymers is efficient and controllable, which offers a facile approach to modulating the structures and performances of UV-curable materials.

## 2. Materials and Methods

### 2.1. Materials

Poly(propylene glycol) (PPG; with a molecular weight of 1000 g/mol; 99%) was purchased from Adams Reagent Co., Ltd. (Shanghai, China). Isophorone diisocyanate (IPDI; 99%) was obtained from Shanghai Merrill Chemical Technology Co., Ltd. (Shanghai, China), and 2-Hydroxypropyl methacrylate (HPMA; 97%) was supplied from Tianjin Zancheng Scientific Co., Ltd. Isobornyl methacrylate (IBOMA; 85–90%) was purchased from Shanghai Maclean Biochemical Technology Co., Ltd., Shanghai, China, Di-n-butyltin dilaurate (DBTDL; chemically pure) was purchased from Sinopharm Chemical Reagent Co., Ltd., Shanghai, China. Hydroquinone (HQ; analytically pure) was obtained from Beijing Xudong Chemical Plant (Beijing, China), and 2-Hydroxy-2-methyl-1-phenylpropan-1-one (PI 1173; 99%) was purchased from Shanghai Yinchang New Materials Co., Ltd. (Shanghai, China). The PPG was dried in a vacuum oven at 80 °C, and the HPMA was dried with anhydrous calcium chloride overnight before use. The other reagents were used as received, without further purification.

### 2.2. Preparation of Prepolymer Resins

The feeding formula for fabricating the UV-curable prepolymer resins is shown in Table 1. To regulate the contents of HIPIH and HIH in the prepolymers, the molar ratios of PPG, IPDI, and HPMA were controlled at 1:2:2, 1:2.5:3, and 1:3:4 for the S1, S2, and S3 prepolymers, respectively. The mass ratio of the prepolymer and IBOMA was fixed at 2.5:1. The total mass of the prepolymer and diluent was 100 g, and the PI 1173 was 5 g.

As an example, the fabrication process of the S1 prepolymer is mentioned below. Firstly, 41.241 g (41.241 mmol) of PPG, 18.311 g (82.482 mmol) of IPDI, and 0.1 g of DBTDL as the catalyst were added into a three-necked round-bottomed flask equipped with a mechanical agitator, an addition tube, and a condenser with a drying tube. The reaction mixture was heated at 60 °C for 2 h. Secondly, 0.05 g of HQ as the inhibitor and 28.571 g of IBOMA as the diluent were added to the flask, and 11.877 g (82.479 mmol) of HPMA was added dropwise for end-capping. After the addition of HPMA, the reaction temperature was elevated to 70 °C. After reacting at 70 °C for 2.5 h, the colorless and transparent reaction mixture was cooled to room temperature, and 5 g of PI 1173 was added dropwise. After thorough mixing and high-speed centrifugation at 6000× *g* rpm for 10 min, the UV-curable prepolymer resin S1 was obtained. The other prepolymer resins, S2 and S3, were fabricated by the same process according to the feeding formula, which is shown in Table 1.

### 2.3. Preparation of Cured Materials

The UV-curable prepolymer resin was slowly poured into a homemade 7 × 7 cm rectangular glass mold (the thickness can be adjusted as needed, about 150~2000 μm) or coated on a tin plate (for adhesion, pencil hardness, and flexibility measurements). Using a 400 W Mercury lamp with a wavelength of 365 nm as the UV source, the curable resin was irradiated for 20 min at ambient temperature, and the distance from the UV lamp to the sample surface was fixed at 15 cm. Here, nearly no sticky cured samples were obtained. For the cured sample characterization, all of the samples were treated at 80 °C for 10 h, except for the FT-IR test.

### 2.4. Characterization

The gel permeation chromatography (GPC) analysis of the prepolymers required a purification procedure. First, a certain amount of petroleum ether was charged into a beaker, after which an appropriate amount of the prepolymer resin (without a PI) was poured into the beaker. Sticky liquids would sink to the bottom. After gently stirring with a glass rod (at about 60 rpm), the IBOMA and traces of impurities in the prepolymer resin would dissolve in the petroleum ether. After keeping the beaker quiet for 10 min, the petroleum ether solution was slowly poured out. After repeating this process five times, the washed sample was put into a vacuum oven and dried at 50 °C for 48 h to obtain a pure viscous prepolymer, which was used for the GPC analysis. The GPC test was performed with an Agilent 1260 Infinity II-MDS instrument. The prepolymer was dissolved in tetrahydrofuran (THF) to a concentration of about 3 mg/mL, with the eluent of THF at a column temperature of 30 °C and a flow rate of 1.0 mL/min. The standard curves were corrected using monodisperse polystyrene with different molecular weights.

In order to obtain a precise and accurate proton nuclear magnetic resonance (^1^H NMR) spectrum, the prepolymer sample used for the ^1^H NMR analysis was prepared separately based on the feeding formula for S2, except that IBOMA was replaced by the volatile solvent butanone. In brief, 7.549 g of PPG, 4.190 g of IPDI, and 0.015 g of DBTDL were added into a three-necked round-bottomed flask equipped with a mechanical agitator, an addition tube, and a condenser with a drying tube. After reacting at 60 °C for 2 h, 0.0075 g of HQ and 3 mL of butanone (which reduces the viscosity) were added to the flask, and 3.261 g of HPMA was added dropwise. Then, the reaction was conducted at 70 °C for 2.5 h. The reaction solution was directly put into a vacuum oven and dried at 50 °C for 24 h. The resulting prepolymer sample was dissolved in deuterated chloroform (CDCl_3_), and the spectrum was recorded on a JEOL 400YH instrument (400 MHz).

The Fourier transmission infrared (FT-IR) spectrum of the liquid sample S2 was recorded on an FT-IR instrument (IR Tracer-100, SHIMADZU Ltd., Kyoto, Japan) using a KBr pellet. The FT-IR spectra of the S2 cured samples were recorded by attenuated total reflection (ATR). The scan range was 400–4000 cm^−1^, with a resolution of 2 cm^−1^.

The morphology of the cured samples was analyzed by a TESCAN VEGA3 scanning electron microscope (SEM) with a 10 KV accelerating voltage after gold plating.

### 2.5. Analysis on Properties

The viscosities of the UV-curable prepolymer resins were measured by an NDJ-1 rotational viscometer (Shanghai, China) at 25 °C after high-speed centrifugation at 6000× *g* rpm for 10 min.

The gel content of the cured samples was tested by a Soxhlet extraction. The small pieces of the cured samples were packed wholly using filter paper in a Soxhlet extractor, using toluene as the solvent to Soxhlet extract at 160 °C for 12 h. After this, the samples were dried in a vacuum oven at 80 °C for 24 h. The gel content (G) was calculated from Equation (1) as follows:

(1) $$G=\frac{\omega_{2}}{\omega_{1}}\times 100\%$$

where ω_1_ and ω_2_ are the masses of the cured samples before the Soxhlet extraction and after the vacuum drying, respectively.

The swelling degree was carried out as follows: pieces of the cured coating were soaked in different solvents for 24 h at room temperature, after which they were taken out and wiped with filter paper to remove the solvent from the surface. The swelling degree (S) was calculated from Equation (2) as follows:

(2) $$S=\frac{m_{2}-m_{1}}{m_{1}}\times 100\%$$

where *m*_1_ and *m*_2_ are the masses of the cured samples before and after soaking, respectively.

The volume shrinkage (V) of the curable resin was measured according to the ISO 3521 standard using a pycnometer. It can be calculated by Equation (3) as follows:

(3) $$V=\frac{1/\rho_{2}-1/\rho_{1}}{1/\rho_{1}}\times 100\%$$

where *ρ*_1_ and *ρ*_2_ are the densities of the resin (complete formulation) before and after curing, respectively. Each experiment was performed at least three times in parallel to obtain the average value.

The gloss of the cured films was measured by a WGG-60 glossmeter (Shanghai, China; the detection range was 0–199 GU) with an incidence angle of 60 for each sample according to the GB/T 4893.6-2013 standard.

The Shore hardnesses of the cured films were determined according to the GB/T 531.1-2008 standard using the HLX-D Shore hardness tester, taking the median after 5 measurements. The adhesion grade of the cured films on the tin plates was determined according to the GB/T 4893.4-2013 standard (the adhesion levels included the following: 0, 1, 2, 3, 4, and 5, from best to worst; the experiments were conducted in three different locations). The pencil hardness was determined according to the GB/T 6739-2006 standard (the hardness levels from hardest to softest were as follows: 6H, 5H, 4H, 3H, 2H, H, HB, B, 2B, 3B, 4B, 5B, and 6B; two parallel measurements were performed; the tests were performed again if inconsistent). The flexibility of the cured films on the tin plates was determined according to the GB/T 1731-2020 standard with a bending tester. The testing plates were bent around cylindrical mandrels, and the flexibility was determined by the diameter of the shaft used (including the following: 15, 10, 5, 4, 3, 2, and 1 mm; the smaller diameter revealed a higher flexibility; the same mandrel was tested three times).

The tensile tests were performed using an Instron 5967 testing machine (Instron Co., Ltd., Norwood, MA, USA) with a gauge length of 20 mm at room temperature. The extension rate was 10 mm/min. The cured samples with a size of 30 × 7 × 0.7 mm^3^ (the exact thickness and width were measured three times by a helical micrometer) were tested to calculate the average values.

The heat behavior of the cured samples (~4 mg) was performed on a NETZSCH DSC 214 Polyma differential scanning calorimeter (DSC). The samples were heated and cooled from 0 °C to 150 °C twice at a heating rate of 20 K/min with a nitrogen flow rate of 60 mL/min.

A dynamic thermo-mechanical analysis (DMA) was performed on a TA instrument DMA 850 using a parallel plate compression clamp. The cured samples were cut into rectangular samples of 30 mm × 7 mm × 0.7 mm. In the oscillation temperature ramp test, the temperature range was 0~140 °C, the heating rate was 3 K/min, and the frequency was 1 Hz.

A thermogravimetric analysis (TGA) was conducted using a TG 209 thermogravimetric analyzer (NETZSCH Corporation, Yokohama, Japan). The TGA was performed from 35 to 900 °C at a heating rate of 10 °C/min under a nitrogen flow rate of 20 mL/min.

## 3. Results and Discussion

### 3.1. Synthesis, UV Curing, and Characterization

**Scheme 1:**
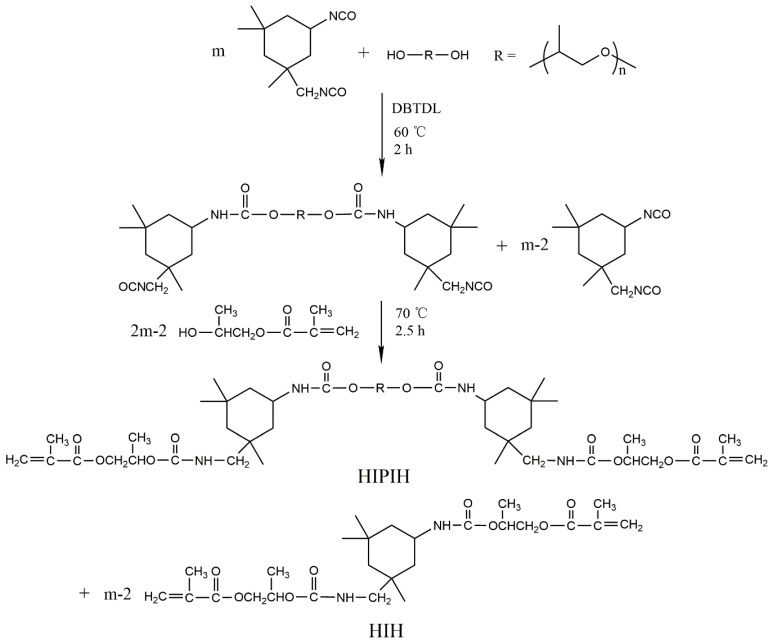
Schematic diagram for the fabrication of the prepolymer molecules; m = 2, 2.5, and 3 for S1, S2, and S3, respectively.

To adjust the structures and performances of the UV-cured materials, a series of polyurethane methacrylate prepolymers was designed and fabricated via a two-step procedure. First, excessive IPDI was reacted with PPG to form an isocyanate-terminated polyurethane prepolymer, which was then fully end-capped by HPMA using IBOMA as the diluent. By simply adjusting the molar ratios between IPDI and PPG, three UV-curable prepolymer resins (defined as S1, S2, and S3) with different contents of HIPIH (with soft PPG segments) and HIH (without a PPG segment) were obtained (see Table 1) after the addition of the photoinitiator PI 1173. According to the molar ratios of IPDI to PPG, the S1 prepolymer should have the highest content of HIPIH and the smallest content of HIH; the S2 prepolymer should have moderate contents of both, while the S3 prepolymer should have the smallest content of HIPIH and the highest content of HIH. Accordingly, the content of methacrylate groups increases in the order of S1, S2, and S3. In the second step of the reaction, nonvolatile IBOMA was included as the reactive diluent to decrease the solution’s viscosity. At the fixed mass ratio of the prepolymer to IBOMA (2.5:1), the viscosity of the prepolymer resins increased slightly from 2700 cp (S1) to 3000 cp (S2) and then further to 3100 cp (S3) (see Table 1). The fabrication process is convenient, controllable, and suitable for large-scale preparations of UV-curable resins. The total fabrication process is outlined in Scheme 1.

**Figure 1:**
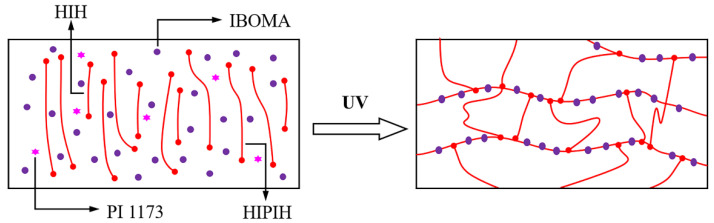
Formation of co-network architecture after UV irradiation of the prepolymer resin.

In the subsequent UV-curing process, as the prepolymer resins were subjected to UV irradiation, the photoinitiator was first excited and disrupted to generate free radicals, which subsequently initiated free-radical copolymerization of the prepolymer and IBOMA. As the polymerization progressed, the reactive components, based on their reactive methacrylate groups, including HIPIH, HIH, and IBOMA, interlinked together to form co-networks, that is, cross-linked polymers, in which the chains are connected with macromolecular cross-linkers [27,28,29]. It is obvious that the higher content of methacrylate groups in the prepolymer gave rise to a tighter co-network architecture. Thus, the co-network became tighter and tighter from the S1 to the S3 cured samples. Figure 1 shows the formation of the co-network after the UV irradiation of the prepolymer resin.

**Figure 2:**
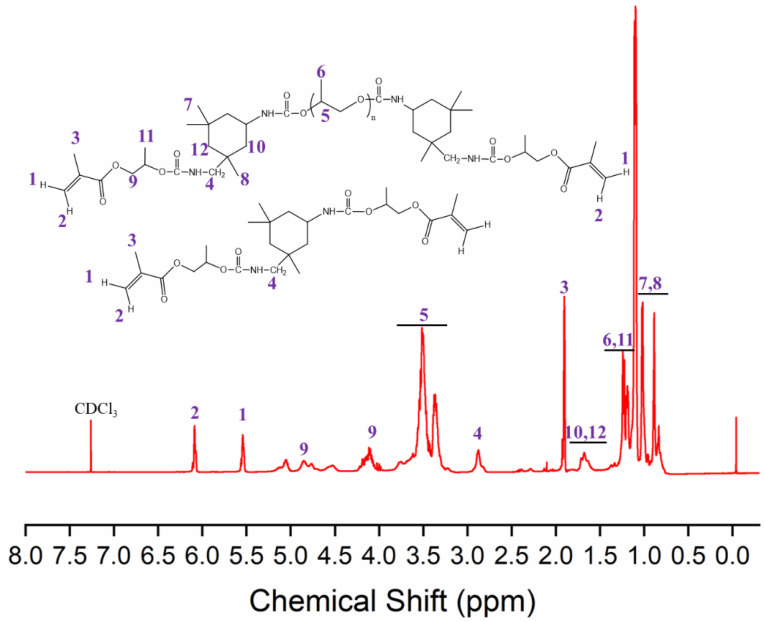
The ^1^H NMR spectrum of the prepolymer mixture.

The prepolymer was further prepared according to the feeding formula for S2, except that butanone was used instead of IBOMA. After the volatilization of butanone from the sample by vacuum drying, the obtained viscous liquid (pure prepolymer) was subjected to nuclear magnetic analysis to understand its molecular structure. The ^1^H NMR spectrum of the prepolymer is shown in Figure 2. The peaks at 6.09 ppm and 5.54 ppm correspond to the two protic chemical shifts of the methacrylate group, respectively (these two peak areas are equal and normalized). The peak at 1.9 ppm corresponds to the methyl protons of the methacrylate group [30], indicating that the methacrylate groups were successfully introduced into the chains after the reaction. The peak at 2.7~3.0 ppm is assigned to the protons of the methylene group linked to an N atom of the carbamate group [23], suggesting the success of the reaction between HPMA and IPDI. Furthermore, the chemical shift of 3.2~3.8 ppm is assigned to the three protons of the methylene group and the tertiary carbon atom in the PPG building unit [31]. The peak at 1.15–1.29 ppm corresponds to the protons of the methyl linked to the PPG chain and HPMA. The peak at 4.10 and 4.84 ppm was assigned to the proton at the methene of the HPMA moiety. The methyl protons of the IPDI moiety had a chemical shift of 0.85–1.04 ppm. The protons at the methene of the IPDI moiety had a chemical shift of 1.56–1.75 ppm [32]. These results indicate that the prepolymer with the desired structure units was formed after the reaction.

**Figure 3:**
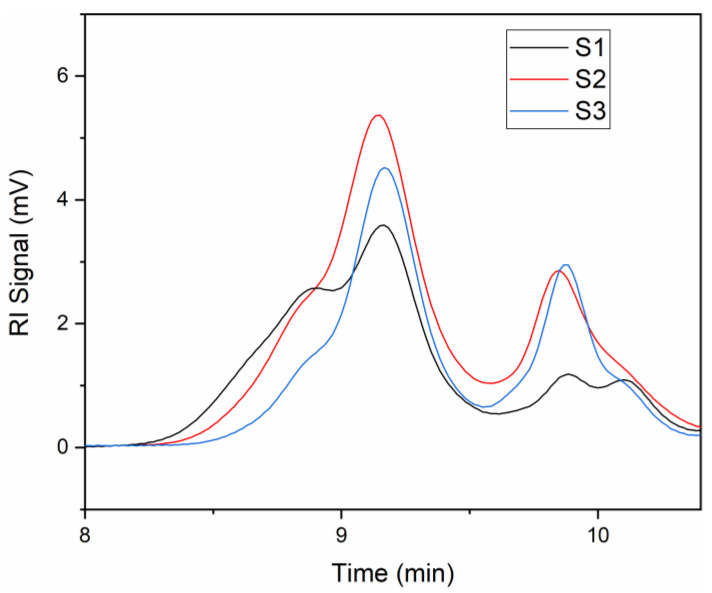
GPC curves of the prepolymers.

According to the idealized prepolymer structures in Scheme 1, HIPIH, corresponding to the structure of HPMA-IPDI-PPG-IPDI-HPMA, has a molar weight of 1732; HIH, with the structure of HPMA-IPDI-HPMA, has a smaller molar weight of 510. Figure 3 shows the GPC chromatograms of the corresponding prepolymers of the three samples, and the relevant values obtained by software are listed in Table 2.

Both the S2 and S3 prepolymers display typical bimodal molecular weight distributions. The peak with a short retention time (of about 9.2 min) corresponds to the HIPIH structure, and the peak with a long retention time (of about 9.9 min) corresponds to the HIH structure. For the S2 prepolymer, HIPIH has an $M_{n}$ of 2911 and a PDI of 1.40, and HIH has an $M_{n}$ of 608 and a PDI of 1.04. For the S3 prepolymer, HIPIH has an $M_{n}$ of 2578 and a PDI of 1.24, and HIH has an $M_{n}$ of 587 and a PDI of 1.08. Whereas, as we can see, the GPC curve of the S1 prepolymer exhibits a multimodal distribution, with two broad peaks at 8.3–9.5 min and one minimal peak at 9.9 min (the peak at 9.9 min corresponds to HIH, and the peak at 10.1 belongs to an impure peak; see Figure S1). This is a deviation from the ideal situation, where only one peak and only the HIPIH structure should exist. The broad peak of the S1 GPC curve at 8.3–9.5 min corresponds to a number-average molar weight ($M_{n}$) of 3060 and a polydispersity index (PDI) of 1.48. From the S1 to the S3 prepolymers, it can be observed that the $M_{n}$ and PDI of HIPIH gradually decreased with the increase in the molar ratios of IPDI to PPG (2 for S1, 2.5 for S2, and 3 for S3), suggesting that the distribution of the molecular chain length became more and more uniform. This is because excessive IPDI can inhibit chain extension. However, the molar ratio of IPDI to PPG is only 2 in the S1 prepolymer; thus, it is inevitable that a certain chain extension reaction would occur in the first step of the reaction, giving rise to polymer molecules with larger molecular weights and higher PDIs. Meanwhile, a small amount of unreacted IPDI would be directly capped by HPMA in the subsequent reaction, resulting in an insignificantly small peak with a retention time of 9.9 min. In fact, for the S1 prepolymer, the peak at 8.9 min corresponded to a higher molecular weight, which resulted from the chain extension reactions of PPG and IPDI; the minimal peak at 9.9 min corresponded to the HIH structure. Conversely, for the S2 and S3 prepolymers, the chain extension was effectively inhibited, and a considerable amount of IPDI remained after the first-step reaction because of their higher IPDI/PPG molar ratios; thus, more IPDI was end-capped directly by HPMA in the second-step reaction, leading to comparatively bigger peaks corresponding to HIH, especially for the S3 prepolymer. The GPC results demonstrate our conjecture about the structures of the prepolymers. Thus, the contents of HIPIH and HIH can be rationally adjusted simply via the feeding ratios of the reactants.

**Figure 4:**
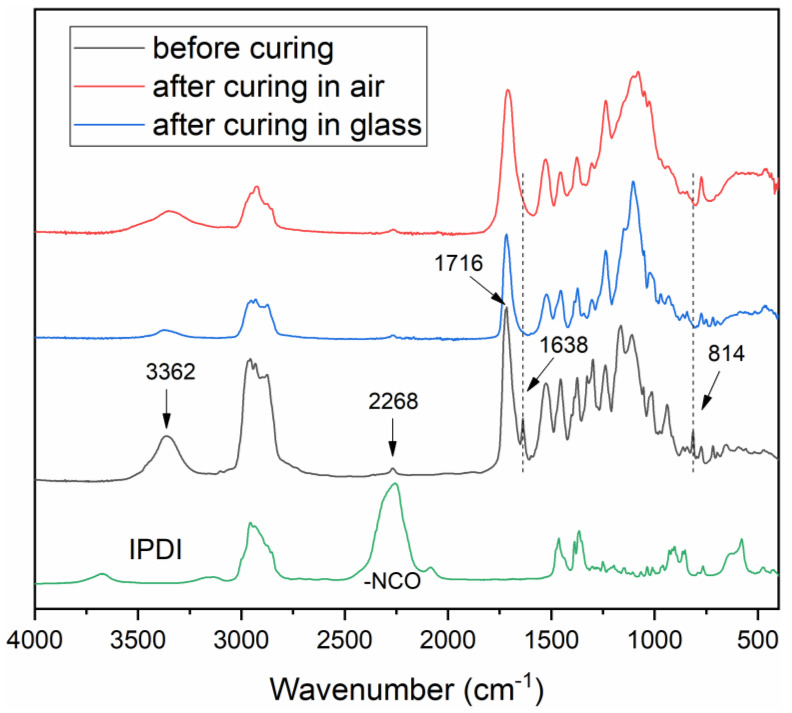
FT-IR spectra of IPDI and the S1 resin before and after UV irradiation.

To understand the UV-curing extent, the S1 prepolymer resin was irradiated by UV light in two ways: one in air and the other in a sealed glass tube. After the irradiation, the resulting cured samples were directly subjected to FT-IR analyses. The FT-IR spectra of the IPDI, S1 prepolymer, and the corresponding UV-cured materials are shown in Figure 4. There is a strong and broad peak at around 2260 cm^−1^ in the IR spectrum of IPDI, which belongs to the stretching vibration peak of the isocyanate group (–N=C=O). From the spectrum of the S1 prepolymer resin, we can see that the negligible peak at 2268 cm^−1^ almost disappeared, indicating that the addition reaction between –NCO and –OH is nearly complete in the synthesis of the prepolymer, similar to the literature results [23]. A broad N–H stretching vibration peak appeared at 3362 cm^−1^, and a C=O stretching vibration peak appeared at 1716 cm^−1^, suggesting the formation of the carbamate group (–NHCOO–). This spectrum also manifests two distinct peaks that appeared at 1638 cm^−1^ and 814 cm^−1^, which correspond to the C=C stretching vibration and the =C–H out-of-plane bending vibration in the methacrylate groups, respectively. This, again, proves that HPMA was successfully attached to the prepolymer molecules, which is consistent with the GPC and ^1^H NMR results. From the spectra of the cured samples, whether the resin was irradiated in air or not, the two characteristic peaks at 1638 cm^−1^ and 814 cm^−1^ disappeared after the UV radiation. Thus, the C=C double bonds had been reacted almost completely in the curing process. Even if the sample exposed to air is subjected to oxygen inhibition, the reaction extent of the C=C double bonds could be quite high, suggesting a high cross-linking degree for the cured sample.

**Figure 5:**
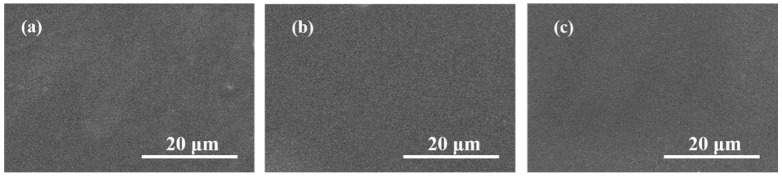
SEM images of the cured films derived from the prepolymers of (**a**) S1; (**b**) S2; (**c**) S3.

SEM measurements are helpful for understanding the surface and internal structures of UV-cured materials [33]. Figure 5 represents the SEM images of the surfaces of the three cured films. It can be seen that all of the surfaces are quite smooth, with almost no cracks, holes, grooves, or bumps observed on them. These microtopography results indicate that all of the components are mixed evenly, and the compatibility of soft and hard segments is good in these systems, although polyurethane may have phase separation because of the existence of soft and hard segments. This also suggests that all of the components containing C=C double bonds effectively participated in the polymerization reaction.

### 3.2. Performances of Cured Materials

Enhancing gel content and solvent resistance can endow the cured samples with reliable mechanical properties. As it can be seen from Table 3, all of the cured films have a clearly distinct gel content and solvent resistance. The S1 cured sample has the lowest gel content of 93.90%, which is still quite a high value. With the increase in the HIH content in the prepolymer, the double bond density per unit volume increases accordingly, which leads to a gradual increase in the cross-linking density and gel content. Various solvents were applied to carry out the swelling experiments, and the corresponding results are listed in Table 3. Compared with the S1 cured sample, which has mostly HIPIH in the prepolymer, the S2 and S3 cured samples showed improved solvent resistances. All of them have rather low water absorption, among which the S1 cured sample has the strongest water absorption with a swelling degree of 1.98%, while the S2 and S3 cured samples have swelling degrees of 1.21% and 1.17%, respectively. The rather low water absorption may suggest the hydrophobic nature of these samples. The solvent resistance of the cured samples is obviously feebler in ethanol or acetone than in pure water, and the swelling degree in these organic solvents significantly decreases from S1 to S3 (see Table 3). These results further explain that the high content of HIH increases the cross-linking density of the co-network, thus, improving the gel content and solvent resistance. Volume shrinkage is an important property of the cured samples. Excessive volume shrinkage will lead to poor adhesion and stress on the fixed devices, resulting in a series of adverse results. As shown in Table 3, the volume shrinkages of the cured samples are all lower than 5%, ranging between 4.5% and 4.8% (S2 has the minimum volume shrinkage of 4.5%), which are very attractive values compared with the literature results [23,33,34]. Since HIH does not have the PPG segment, it can provide the cured sample with a high density of the carbamate groups as hard segments, thus, endowing the material with rigidity. A prepolymer with a smaller molecular weight will be encountered with a higher volume shrinkage; conversely, a larger molecular weight may generate problems, such as lower cross-linking density and poorer solvent resistance. Besides, all of the cured materials are transparent with a rather high gloss (see Table 3).

**Figure 6:**
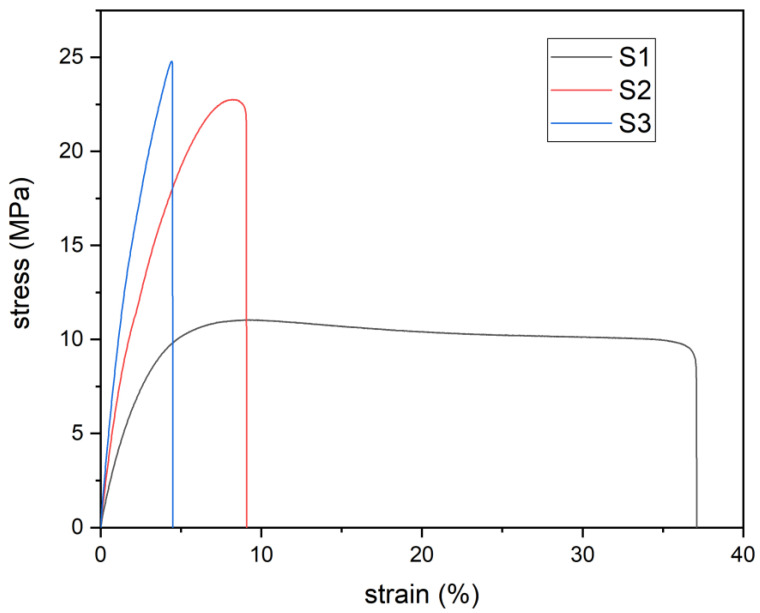
Stress and strain curves of the cured samples.

The mechanical performances of the cured films are shown in Table 4. The Shore hardness increases significantly from 66 for the S1 cured film to 76 for the S2 cured film and further to 82 for the S3 cured film. This is because the content of soft polyether chains is reduced, while the content of rigid HIH increases from the S1 to the S3 cured sample; therefore, the hydrogen bond aggregation domains become more abundant and the cross-linking density increases. The pencil hardness results are consistent with the Shore hardness results. Both the cured films of S1 and S2 have high adhesions (level 0) on the tin plate, while the adhesion of the S3 cured film markedly declines to level 3. We can deduce that, as for improving the adhesion level, it is advantageous to have a certain amount of flexible PPG segments in the matrix. The stiffness and brittleness of the S3 cured sample with the least PPG soft segments are so high that the sample is unable to resist exterior forces, thus, decreasing the adhesion on the tin plate. As the stress and strain curves in Figure 6 show, the modulus and tensile strength increase with more HIH in the prepolymers. The S1 cured sample with the most HIPIH is soft and ductile; its Young’s modulus and tensile strength are 410.8 MPa and 11.1 MPa, respectively, while the S2 and S3 cured samples containing more HIH display a significant enhancement in modulus and tensile strength. The elongation at break reached 37.1% for the S1 cured sample, and it decreased to 9.1% for the S2 cured sample, after which it declined further to 4.5% for the S3 cured sample. The trend of elongation at break is contrary to the modulus, hardness, and tensile strength, which also proves that the cross-linking density is constantly improving from the S1 to the S3 cured samples. Based on the stress–strain curves, the S1 and S2 cured samples underwent a ductile fracture (after the yield point), whereas a brittle fracture (before the yield point) happened on the S3 cured sample. There are obvious differences in the nature of the S1 and S2 cured samples, although they both break after the yield point. The S1 cured sample can still be stretched a lot after the yield point, with an elongation of up to 37.1%, while the S2 cured sample broke shortly after the yield point. As for the S3 cured sample, it fails before the yield point, corresponding to minimum deformation. The yield stress is observed to increase with the content of HIH. From S1 to S3, the mechanical properties of the cured samples experience a transition from “ductile” to “brittle”, which can be attributed to the higher cross-linking density and the more rigid carbamate groups in the matrices. This ductile-to-brittle transition and the increase in the cross-linking density have also been reported in the literature [35].

### 3.3. Thermo Properties

**Figure 7:**
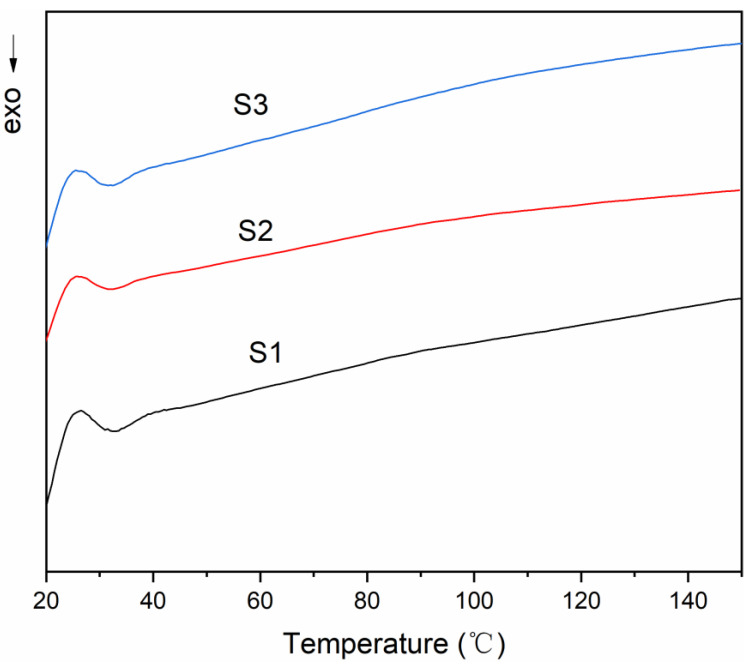
The second heating DSC curves of the three cured samples.

Glass transition temperature (Tg) refers to the temperature corresponding to the transition of a polymer from a glass state to a high elastic state. As mentioned above, there may be phase separation between the soft and hard segments in polyurethane; the polyether segment constitutes the soft segment, and the carbamate groups contain abundant hydrogen bonds with strong polarity, forming the hard segment, which may lead to two different phase transition temperatures in the polyurethane system. We previously carried out a DSC experiment on S1 at a temperature ranging from −70 to 150 °C (see Figure S2), but no obvious transition was observed in the whole temperature region. For this reason, we afterwards carried out the DSC experiments at a higher temperature range (0–150 °C). Figure 7 shows that the three cured samples have similar DSC curves. However, all of them do not manifest the typical phase transition temperature but express one relatively gentle transition region. Thus, it was not possible to obtain the accurate Tgs based on these curves. We suppose that the thermal effect of these UV-cured samples with quite a high cross-linking density is insensitive because of the restricted segment motion. Thus, DMA experiments were carried out to obtain accurate and reliable results about the thermal behavior of the cured samples.

**Figure 8:**
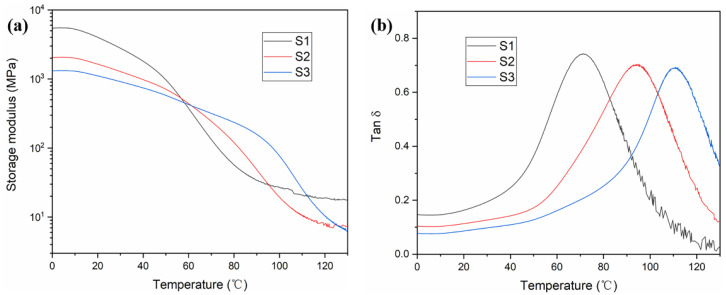
(**a**) Storage modulus and (**b**) loss factor of the cured samples.

The thermo-mechanical properties of the cured samples were characterized by DMA. The measuring curves and the related results are shown in Figure 8 and Table 5, respectively. It can be seen from Figure 8a that at a wide range of low temperatures, the storage moduli of the three samples are basically maintained at high values (>1000 MPa). As the temperature increases further, the storage modulus decreases continuously. When the temperature passes through the transition region of the Tg, the modulus decreases promptly until a new plateau is reached at an even higher temperature. The storage moduli at 25 °C, which are listed in Table 5 as E’_25_, decrease in order of the S1 cured sample (3358.7 MPa), S2 cured sample (1453.7 MPa), and S3 cured sample (1011.2 MPa), opposite to the gel content, hardness, and Young’s modulus listed in Table 3 and Table 4. The differences in the moduli of these samples from the DMA and tensile mechanics tests should be attributed to the different measuring conditions. Generally, the S3 cured sample is the most rigid and the relaxation time is the shortest, while the S1 cured sample is the softest and the relaxation time is the longest. The S2 cured sample is in between. Under the DMA testing condition, for the S3 cured sample with the least relaxation time, its molecular chain motion is fully released. However, the “softest” S1 cured sample cannot keep up with the measuring frequency of 1 Hz, so it exhibits the highest storage modulus among them. In the loss factor curve, the peaks correspond to the Tgs of the cured samples. As indicated in Figure 8b and Table 5, the Tgs measured by the DMA are 71.2 °C, 94.2 °C, and 110.9 °C for the S1, S2, and S3 cured samples, respectively. It is evident that the Tgs of the cured samples increase upon increasing the content of HIH in the prepolymers. This result further indicates that the high content of HIH can improve the cross-linking density. In fact, when the service temperature of the polymer matrices exceeds their Tgs, they will rapidly lose their mechanical performance, including their strength and stiffness [36]. The DMA experiments show that these systems, especially the S2 and S3 cured materials, have Tgs at around 100 °C, far exceeding room temperature and service temperature in most cases. Thus, the samples with high Tgs have good heat resistance and can maintain their stable mechanical properties over a wide temperature range. Only one obvious peak is observed in these curves, suggesting good compatibility between these systems. The thermo-mechanical properties of the cured films are closely related to the cross-linking density. The results indicate that changing the ratio of HIH to HIPIH (or the hard segments to the soft segments) has a significant effect on the thermo-mechanical properties of the cured systems. The high and stable storage moduli at around 25 °C, together with the rather high Tgs, should make these samples stiff enough and less deformable when used. These merits should make them suitable for use in events requiring high and stable mechanical properties.

**Figure 9:**
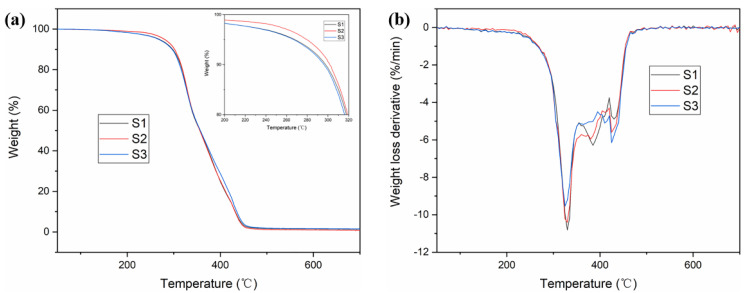
(**a**) TGA curves and (**b**) DTG curves of the cured samples.

In order to make UV-cured samples with good performance in complex and changeable climates, thermal stability is an important factor that should be considered. The three TGA curves and the derivative thermogravimetry (DTG) curves are shown in Figure 9, and their important temperature parameters are listed in Table 5. The TGA curves of the three samples are quite similar, and all of them have an initial decomposition temperature (5% weight loss) higher than 260 °C. According to the DTG curve of the S1 cured sample, the degradation process can be divided into three steps. The first pyrolysis process (225~355 °C) mainly corresponds to the carbamate region that forms the hard segment; the second pyrolysis process (355~420 °C) corresponds to the decomposition of the polyether segments in the polymer chains, and the third pyrolysis step (420~500 °C) is attributed to the gradual degradation of the char residue [37]. The DTG curve shows that the peak corresponding to the first degradation process is the most obvious. The pyrolysis of the S2 and S3 cured samples can also be divided into three similar steps, but the second peak is not so obvious, which may be due to the reduced contents of the soft polyether segments. It is interesting to note that the S2 cured sample has the highest initial decomposition temperature of 279.6 °C. It is speculated that the S2 cured sample possesses a larger cross-linking density relative to the S1 cured sample, which is conducive to thermal stability. Meanwhile, the quite high content of stable PPG (compared to the S3 cured sample) also plays a positive role, giving the S2 cured sample the best thermal resistance. The temperatures at the maximum rate of weight loss are 332.5 °C, 327.2 °C, and 325.8 °C for the S1, S2, and S3 cured samples, respectively. These results demonstrate the excellent thermal stability of the cured samples. In addition, there is little difference between the three pyrolysis curves, suggesting that changing the contents of HIPIH and HIH in the samples will not greatly affect their thermostability.

## 4. Conclusions

In summary, UV-curable polyurethane acrylate prepolymers with different contents of HIPIH and HIH were successfully fabricated via adjusting the feeding formula of the raw materials via a one-pot reaction process. This solvent-free fabrication process is simple, convenient, and controllable. The prepolymer structures were confirmed by various techniques. The UV radiation of the prepolymer resins produced high-performance cured materials with high gel contents and solvent resistances, low volume shrinkages, high glosses, excellent mechanical properties, and high thermal stabilities. By increasing the content of HIH in the prepolymers, the Shore hardness, Young’s modulus, and the tensile strength of the cured films increased, whereas the elongation at break decreased accordingly. DMA analyses indicated that the cured samples possessed high and stable storage moduli at the service temperature of around 25 °C, which is far below the Tgs. By simply adjusting the contents of HIPIH and HIH in the prepolymers via feeding composition, the performances of the UV-cured materials could be modulated effectively, adapting to diverse application requirements.

## Figures and Tables

**Table 1:** Feeding formula and viscosity for the UV-curable prepolymer resins.

Sample	PPG (g; mmol)	IPDI (g; mmol)	HPMA (g; mmol)	IBOMA (g; mmol)	PI 1173 (g)	HQ (g)	DBTDL (g)	Viscosity (cp)
S1	41.241	18.311	11.877	28.571	5	0.05	0.1	2700
	41.241	82.482	82.479	128.70				
S2	35.948	19.951	15.530	28.571	5	0.05	0.1	3000
	35.948	89.869	107.847	128.70				
S3	31.859	21.218	18.351	28.571	5	0.05	0.1	3100
	31.859	95.577	127.438	128.70				

**Table 2:** GPC results of the prepolymers.

Sample	Results at Short Retention Time			Results at Long Retention Time		
	$M_{n}\;(g/\mathrm{mol})$	${M_{w}}^{1}\;(g/\mathrm{mol})$	PDI	$M_{n}\;(g/\mathrm{mol})$	$M_{w}\;(g/\mathrm{mol})$	PDI
S1	3060	4532	1.48	--	--	--
S2	2911	4071	1.40	608	633	1.04
S3	2578	3198	1.24	587	633	1.08

^1^ weight-average molar weight.

**Table 3:** Gel content, swelling degree, volume shrinkage, and gloss of the cured materials.

Sample	Gel Content (%)	Swelling Degree (%)			Volume Shrinkage (%)	Gloss
		Water	Ethanol	Acetone		
S1	93.90	1.98	50.22	57.50	4.8	142.0
S2	96.98	1.21	31.45	45.99	4.5	138.6
S3	97.84	1.17	25.55	39.25	4.7	133.0

**Table 4:** Mechanical properties of the cured samples.

Sample	Shore Hardness	Pencil Hardness	Adhesion Level	Flexibility (mm)	E ^1^ (MPa)	σ^2^ (MPa)	ε^3^ (%)
S1	66	HB	0	1	410.8	11.1	37.1
S2	76	2H	0	1	697.0	22.8	9.1
S3	82	3H	3	1	994.9	24.8	4.5

^1^ Young’s modulus; ^2^ Tensile strength; ^3^ Strain at break.

**Table 5:** Thermal properties of the cured samples.

Sample	Tg ^1^ (°C)	E’_25_ ^2^ (MPa)	T_5%_ ^3^ (°C)	T_p_ ^4^ (°C)
S1	71.2	3358.7	268.1	332.5
S2	94.2	1453.7	279.6	327.2
S3	110.9	1011.2	266.8	325.8

^1^ Tg measured by DMA, ^2^ Storage modulus at 25 °C, ^3^ Onset decomposition temperature at 5% weight loss; ^4^ The temperature at the maximum rate of weight loss.

## Data Availability

The data presented in this study are available in the article and Supplementary Materials.

## Supplementary Materials

The following supporting information can be downloaded at: https://www.mdpi.com/article/10.3390/ma15238586/s1, Figure S1: GPC curves of S1, blank control (only THF), and IBOMA; Figure S2: DSC curve of S1 at the temperature region from −70 to 150 °C.

## References

[1] Lang M., Hirner S., Wiesbrock F., Fuchs P. (2022). A Review on Modeling Cure Kinetics and Mechanisms of Photopolymerization. Polymers.

[2] Zhou J., Allonas X., Ibrahim A., Liu X. (2019). Progress in the development of polymeric and multifunctional photoinitiators. Prog. Polym. Sci..

[3] Gao X., Yuan W., Yang Y., Wu Y., Wang C., Wu X., Zhang X., Yuan Y., Tang Y., Chen Y. (2022). High-Performance and Highly Safe Solvate Ionic Liquid-Based Gel Polymer Electrolyte by Rapid UV-Curing for Lithium-Ion Batteries. ACS Appl. Mater. Interfaces.

[4] Czachor-Jadacka D., Pilch-Pitera B. (2021). Progress in development of UV curable powder coatings. Prog. Org. Coat..

[5] Zhao D., Liu S., Wu Y., Guan T., Sun N., Ren B. (2019). Self-healing UV light-curable resins containing disulfide group: Synthesis and application in UV coatings. Prog. Org. Coat..

[6] Wang Z.Y., Liang H.B., Yang H.T., Xiong L., Zhou J.P., Huang S.M., Zhao C.H., Zhong J., Fan X.F. (2019). UV-curable self-healing polyurethane coating based on thiol-ene and Diels-Alder double click reactions. Prog. Org. Coat..

[7] Hakeim O.A., Arafa A.A., Zahran M.K., Abdou L.A.W. (2018). Characterisation and application of pigmented UV-curable inkjet inks. Pigment. Resin Technol..

[8] Saleh E., Woolliams P., Clarke B., Gregory A., Greedy S., Smartt C., Wildman R., Ashcroft I., Hague R., Dickens P. (2017). 3D inkjet-printed UV-curable inks for multi-functional electromagnetic applications. Addit. Manuf..

[9] Kowalczyk K., Kowalczyk A. (2015). UV-curable epoxy varnishes modified with polyvinyl resins. Prog. Org. Coat..

[10] Feng X., Li G. (2022). UV curable, flame retardant, and pressure-sensitive adhesives with two-way shape memory effect. Polymer.

[11] Lim W.B., Bae J.H., Seo M.J., Min J.G., Lee J.H., Jung Y.S., Huh P. (2022). A novel UV-curable acryl-polyurethane for flexural 3D printing architectures. Addit. Manuf..

[12] Wang X., Feng Y., Zhang L., Protsak I., Jamali R., Shu Y., Pal P., Wang Z., Yang J., Zhang D. (2020). Fast-cured UV-LED polymer materials filled with high mineral contents as wear-resistant, antibacterial coatings. Chem. Eng. J..

[13] Noe C., Iannucci L., Malburet S., Graillot A., Sangermano M., Grassini S. (2021). New UV-Curable Anticorrosion Coatings from Vegetable Oils. Macromol. Mater. Eng..

[14] Sangermano M., Razza N., Crivello J.V. (2014). Cationic UV-Curing: Technology and Applications. Macromol. Mater. Eng..

[15] Andrzejewska E., Linden L.A., Rabek J.F. (1997). Modelling the kinetics of photoinitiated polymerization of di(meth)acrylates. Polym. Int..

[16] Hermann A., Burr D., Landry V. (2021). Comparative study of the impact of additives against oxygen inhibition on pendulum hardness and abrasion resistance for UV-curable wood finishes. Prog. Org. Coat..

[17] Ligon S.C., Husar B., Wutzel H., Holman R., Liska R. (2014). Strategies to Reduce Oxygen Inhibition in Photoinduced Polymerization. Chem. Rev..

[18] Wang Q., Huang B., Wei X., Shen H. (2015). Study on Shrinkage of Cured Volume for UV-Curing Coatings. Appl. Mech. Mater..

[19] Decker C. (2005). New developments in UV radiation curing of protective coatings. Surf. Coat. Int. Part B Coat. Trans..

[20] Malik M.S., Schloegl S., Wolfahrt M., Sangermano M. (2020). Review on UV-Induced Cationic Frontal Polymerization of Epoxy Monomers. Polymers.

[21] Fu J., Wang L., Yu H., Haroon M., Haq F., Shi W., Wu B., Wang L. (2019). Research progress of UV-curable polyurethane acrylate-based hardening coatings. Prog. Org. Coat..

[22] Jiao X., Liu J., Jin J., Cheng F., Fan Y., Zhang L., Lai G., Hua X., Yang X. (2021). UV-cured transparent silicone materials with high tensile strength prepared from hyperbranched silicon-containing polymers and polyurethane-acrylates. ACS Omega.

[23] Li P., Chu Z., Chen Y., Yuan T., Yang Z. (2021). One-pot and solvent-free synthesis of castor oil-based polyurethane acrylate oligomers for UV-curable coatings applications. Prog. Org. Coat..

[24] Cheng F., Fan Y., He N., Song Y., Shen J., Gong Z., Tong X., Yang X. (2022). Castor oil based high transparent UV cured silicone modified polyurethane acrylate coatings with outstanding tensile strength and good chemical resistance. Prog. Org. Coat..

[25] Noreen A., Mahmood S., Khalid A., Takriff S., Anjum M., Riaz L., Ditta A., Mahmood T. (2022). Synthesis and characterization of bio-based UV curable polyurethane coatings from algal biomass residue. Biomass Convers. Biorefin..

[26] Czech Z., Kabatc J., Bartkowiak M., Mozelewska K., Kwiatkowska D. (2020). Influence of an Alkoxylation Grade of Acrylates on Shrinkage of UV-Curable Compositions. Polymers.

[27] Pasztor S., Becsei B., Szarka G., Thomann Y., Thomann R., Muehlhaupt R., Ivan B. (2020). The Scissors Effect in Action: The Fox-Flory Relationship between the Glass Transition Temperature of Crosslinked Poly(Methyl Methacrylate) and Mc in Nanophase Separated Poly(Methyl Methacrylate)-l-Polyisobutylene Conetworks. Materials.

[28] Fodor C., Stumphauser T., Thomann R., Thomann Y., Ivan B. (2016). Poly(*N*-vinylimidazole)-l-poly(propylene glycol) amphiphilic conetworks and gels: Molecularly forced blends of incompatible polymers with single glass transition temperatures of unusual dependence on the composition. Polym. Chem..

[29] Stumphauser T., Kasza G., Domjan A., Wacha A., Varga Z., Thomann Y., Thomann R., Pasztoi B., Troetschler T.M., Kerscher B. (2020). Nanoconfined Crosslinked Poly(ionic liquid)s with Unprecedented Selective Swelling Properties Obtained by Alkylation in Nanophase-Separated Poly(1-vinylimidazole)-l-poly(tetrahydrofuran) Conetworks. Polymers.

[30] Wu Q., Hu Y., Tang J., Zhang J., Wang C., Shang Q., Feng G., Liu C., Zhou Y., Lei W. (2018). High-performance soybean-oil-based epoxy acrylate resins:“Green” synthesis and application in UV-curable coatings. ACS Sustain. Chem. Eng..

[31] Prabhakar A., Chattopadhyay D., Jagadeesh B., Raju K. (2005). Structural investigations of polypropylene glycol (PPG) and isophorone diisocyanate (IPDI)-based polyurethane prepolymer by 1D and 2D NMR spectroscopy. J. Polym. Sci. Part A Polym. Chem..

[32] Romero-Sabat G., Angel Granda L., Medel S. (2022). Synthesis of UV-curable polyurethane-acrylate hybrids with tuneable hardness and viscoelastic properties on-demand. Mater. Adv..

[33] Xu J., Jiang Y., Zhang T., Dai Y., Yang D., Qiu F., Yu Z., Yang P. (2018). Synthesis of UV-curing waterborne polyurethane-acrylate coating and its photopolymerization kinetics using FT-IR and photo-DSC methods. Prog. Org. Coat..

[34] Yang Z., Shan J., Huang Y., Dong X., Zheng W., Jin Y., Zhou W. (2021). Preparation and mechanism of free-radical/cationic hybrid photosensitive resin with high tensile strength for three-dimensional printing applications. J. Appl. Polym. Sci..

[35] Fei M., Liu T., Zhao B., Otero A., Chang Y.-C., Zhang J. (2021). From Glassy Plastic to Ductile Elastomer: Vegetable Oil-Based UV-Curable Vitrimers and Their Potential Use in 3D Printing. ACS Appl. Polym. Mater..

[36] Sun B., Yang K., Lei Q., Shi H., Li Y., Hu N., Fu S. (2018). High residual mechanical properties at elevated temperatures of carbon fiber/acetylene-functional benzoxazine composite. Compos. Part A Appl. Sci. Manuf..

[37] Ge Q., Wang H., She Y., Jiang S., Cao M., Zhai L., Jiang S. (2015). Synthesis, characterization, and properties of acrylate-modified tung-oil waterborne insulation varnish. J. Appl. Polym. Sci..

