# Supplementary material for: Synthesis and Structure of 2-Hydroxypropyl Methacrylate-Capped Isophorone Diisocyanate and Poly(Propylene Glycol) Urethane Mixtures and the Properties of their UV-Cured Co-Networks with Isobornyl Methacrylate
Source: Materials (Basel). 2022 Dec 1;15(23):8586. doi: 10.3390/ma15238586 (PMC9737471; doi:10.3390/ma15238586)
Supplement: Supplementary file 1 [file materials-15-08586-s001.zip › materials-1981726-supplementary.pdf]

Supporting Information

Synthesis, structure of 2-hydroxypropyl-capped isophorone diisocyanate and poly(propylene glycol) urethane mixtures and the properties of their UV-cured conetworks with isobornyl methacrylate

Junhao Zhou, Liming Tang *

Key Laboratory of Advanced Materials of Ministry of Education of China, Department of Chemical Engineering, Tsinghua University, Beijing 100084, China.

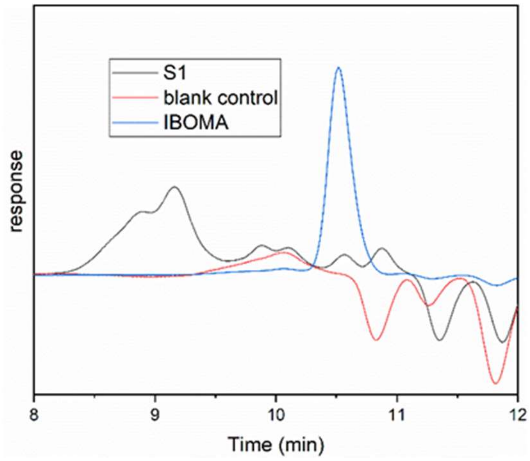

Figure S1. GPC curves of S1, blank control (only THF), and IBOMA.

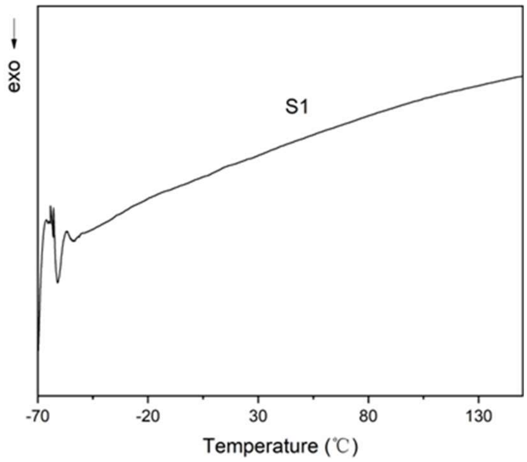

Figure S2. DSC curve of S1 at the temperature region from -70 to 150 °C.
